# The Effect of Erosion on Optical Properties and Surface Texture of Stained Lithium Disilicate Restorations

**DOI:** 10.1155/ijod/8854731

**Published:** 2025-10-13

**Authors:** Fateme Abdarjouy, Farideh Gerami-Panah, Leila Sedighpour, Sima Shahabi

**Affiliations:** ^1^School of Dentistry, Tehran University of Medical Sciences, Tehran, Iran; ^2^Department of Prosthodontics, School of Dentistry, Tehran University of Medical Sciences, Tehran, Iran; ^3^Laser Research Center of Dentistry, Dentistry Research Institute, Tehran University of Medical Sciences, Tehran, Iran; ^4^Department of Dental Biomaterials, School of Dentistry, Tehran University of Medical Sciences, Tehran, Iran

**Keywords:** color stability, erosion, gastric acid, lithium disilicate ceramics, optical properties, translucency

## Abstract

**Objective:**

To evaluate the surface topography, color stability, and translucency of stained lithium disilicate ceramics after immersion in simulated gastric acid.

**Materials and Methods:**

Twenty specimens were prepared from Amber Mill: HASS lithium disilicate ceramic blocks and divided into two groups (*n* = 10). One group was stained and glazed using IPS Ivocolor Essence (Ivoclar Vivadent, Liechtenstein) and the other with GC Initial IQ lustre paste (GC, Japan). Specimens were immersed in 5 mL of 0.113% hydrochloric acid for 96 h at 37°C. morphological changes were evaluated using scanning electron microscopy (SEM), surface roughness was measured with a contact profilometer, and color and translucency were assessed using a spectrophotometer before and after acid immersion.

**Results:**

Acid exposure significantly affected the color of both groups (*p*-value < 0.001), with greater changes observed in the IPS Ivocolor group. Surface roughness parameters (*R*_a_, *R*_q_,  and *R*_z_) also increased significantly in this group following immersion. Translucency parameters remained unchanged in both groups.

**Conclusion:**

Simulated gastric acid adversely affected the color and surface integrity of lithium disilicate ceramics stained with IPS Ivocolor more than those treated with GC Initial Spectrum. The latter demonstrated greater resistance to acidic conditions and may offer better long-term performance in patients at risk of intrinsic acid exposure.

## 1. Introduction

An increase in elderly population in industrialized countries has resulted in distinct oral health problems, including those affecting restorative dentistry such as dental erosion [1, 2]. Dental erosion is defined as the progressive loss of tooth structure by chemical processes that do not involve bacterial action, producing defects that are wedge-shaped depressions often in occlusal, facial and cervical areas by glossary of prosthodontics [3]. Numerous intrinsic and extrinsic factors can contribute to dental erosion. Extrinsic factors include low-pH foods, drinks and also medications such as aspirin or some dental products such as bleach [4, 5]. The main intrinsic factor is gastric acid, which has a lower pH and accordingly a more detrimental effect on teeth than extrinsic factors [6].

Gastric acid can come into contact with teeth in different medical conditions including bulimia nervosa and gastroesophageal reflux disease (GERD). GERD is defined as a condition in which stomach acid and contents back up into the esophagus, producing symptoms of heartburn or regurgitation [7]. It is a highly prevalent disorder, as a recent systematic review has reported a prevalence of 13.98 % with approximately 1.03 billion individuals suffering from the condition globally [8]. Bulimia nervosa is a serious eating disorder characterized by compulsive overeating usually followed by self-induced vomiting or laxative or diuretic abuse. According to Van Eeden et al. [9] up to 3% of females and > 1% of males suffer from this disorder during their lifetime.

Due to the rise in erosive tooth wear, dental rehabilitation for this group of patients, aiming to restore both esthetic and function, has become a subject of interest in recent years. Based on the severity of defects, both direct and indirect restorations can be used in these patients [10]. Regarding indirect restorations, ceramics are popular materials for providing good esthetic, high wear resistance, and optimal biocompatibility. Although ceramics are considered chemically inert biomaterials, they may be affected by low pH with different intensities [1]. The knowledge of how ceramics react to acid can be beneficial for dentists in selecting appropriate restorative material for such patients mentioned above. Previous studies have investigated the impact of acids and specifically gastric acid on physical and mechanical properties of ceramics [1, 4, 11–21]. Yet, according to authors' knowledge, few studies have evaluated the effect of low pH on stained ceramics [19], whereas staining is a commonly used method of simulating natural tooth appearance in ceramic restorations and it has been consequently suggested to evaluate the effect of an acidic environment on the stability of such stains in previous studies [13, 20].

As a result, this study was designed to determine the influence of simulated gastric acid on color, translucency, surface roughness, and surface morphology of stained lithium disilicate glass ceramics. Our null hypothesis was that simulated gastric acid would not affect optical properties and surface texture of stained ceramics.

## 2. Material and Methods

In this study a lithium disilicate–based ceramic, Amber Mill (HASS, Korea) and 2 universal types of stain and glaze including IPS Ivocolor Essence stain and IPS Ivocolor glaze powder (Ivoclar Vivadent, Liechtenstein) and GC Initial IQ lustre paste stain and Gc Initial glaze (GC, Japan) were selected.

### 2.1. Sample Size Calculation

Sample size was calculated to be minimum of 10 ceramic samples per group, according to a previous study assuming *α* = 0.05, *β* = 0.2 and study power of 80% using ANOVA power analysis option of SPSS software [13].

### 2.2. Specimen Preparation

Ceramic blocks were mounted in polyester and cut into disk shaped specimens using a diamond-coated cutting disk (Mecatome T201A, Presi, France) under water cooling. The specimens were crystalized according to manufacturer's instruction to obtain low translucency ceramic disks. One side of each specimen was polished under water cooling using 600, 1000, and 2000 grit silicon carbide papers and a polishing machine. The specimens were checked by a digital caliper (Mitutoyo, Japan) to have dimensions of 12 mm  × 14 mm × 1.15 ± 0.01 mm. then they were cleaned ultrasonically for 10 min and air-dried for 30 s.

The 20 specimens were divided into two groups of 10 according to the stain type. The first group was stained with Ivocolor Essence (E13 espresso) and glazed with IPS Ivocolor glaze powder mixed with IPS Ivocolor Mixing Liquid longlife. For the second group staining was done using GC Initial IQ lustre paste (L-C) followed by glazing with GC initial glaze. For both groups staining and glazing were performed in two separate steps by an experienced operator using bristle brushes; first, they were stained and sintered and secondly, they were glazed and fired according to the manufacturer's instructions.

### 2.3. Aging Protocol

Hunt and MC Intyre's method [22] was used for aging protocol to simulate oral condition in GERD patients. Each specimen was immersed in 5 mL of 0.06 M hydrochloric acid (0.113% solution in deionized water, pH = 1.2) for 96 h in a 37°C incubator (Memmert, Germany) with the stained surface facing up. pH was controlled every 24 h using a pH meter. After aging, the specimens were ultrasonically cleaned in distilled water for 10 min and air-dried.

### 2.4. Optical Measurements

To evaluate the color and translucency, a spectrophotometer (i1 PRO Rev E, X-Rite Co, USA) was used according to CIE LAB (Δ*E*_ab_) and CIEDE2000 (Δ*E*_00_). *L*^*∗*^, *a*^*∗*^, and *b*^*∗*^ were measured for the stained surfaces before and after immersion over a white background. The measurements were repeated three times by the same operator as close as possible to the center of each specimen and the average values were used to calculate total color change. Δ*E*_*ab*_ and Δ*E*_00_ were calculated using the following equations:

  $$\begin{array}{c} \Delta E_{\text{ab}}={\left(\Delta L\right)}^{*}2+{\left(\Delta a\right)}^{*}2+{\left.{\left(\Delta b\right)}^{*}2|\text{⁣}\right)}^{1/2}, \end{array}$$

where Δ*L*^*∗*^ = the difference in lightness, Δ*a*^*∗*^ = red/green axis, and Δ*b*^*∗*^ = yellow/blue axis. Δ*E*_ab_ values were then classified as [23], Δ*E*_ab_ ≤ 1.2 undetectable color changes for human eye, Δ*E*_ab_ > 1.2 the color change detected visually, but acceptable and Δ*E*_ab_ ≥ 2.7 the color change that's not clinically acceptable.

  $$\begin{array}{c} \Delta E_{\text{00}}={\left[{\left(\frac{\Delta L^{\prime }}{K_{\text{L}}S_{\text{L}}}\right)}^{2}+{\left(\frac{\Delta C^{\prime }}{K_{\text{C}}S_{\text{C}}}\right)}^{2}+{\left(\frac{\Delta H^{\prime }}{K_{\text{H}}S_{\text{H}}}\right)}^{2}+R_{\text{T}}\left(\frac{\Delta C^{\prime }}{K_{\text{C}}S_{\text{C}}}\right)+\left(\frac{\Delta H^{\prime }}{K_{\text{H}}S_{\text{H}}}\right)\right]}^{\frac{1}{2}}, \end{array}$$

where Δ*L*′ = difference in lightness, Δ*C*′ = difference in chroma, Δ*H*′ = difference in hue and *K*_L_ : *K*_C_ : *K*_H_ = 1 : 1 : 1.

Δ*E*_00_ values were then classified as [23]: Δ*E*_00_ ≤ 0.8 imperceptable color changes, Δ*E*_00_ > 0.8 the color change perciptible, but acceptable, and Δ*E*_00_ ≥ 1.8 the color change that is not clinically acceptable.

The translucency was evaluated by comparing the light reflectance through each specimen over a black and a white background. TP was obtained using the following formula in which *b* and *w*, respectively, refer to the values against black and white background.

  $$\begin{array}{c} \text{TP}={\left\{{\left(L_{\text{b}}^{*}-L_{\text{w}}^{*}\right)}^{2}+{\left(a_{\text{b}}^{*}-a_{\text{w}}^{*}\right)}^{2}+{\left(b_{\text{b}}^{*}-b_{\text{w}}^{*}\right)}^{2}\right\}}^{\frac{1}{2}}. \end{array}$$

### 2.5. Surface Roughness

To assess surface roughness, *R*_a_ (the arithmetic average of the absolute values of the roughness profile ordinates [24]), *R*_q_ (the square root of the average of the square of the deviation of scan from the mean line [24]), and *R*_z_ (the height difference between the highest and lowest point of the surface) were measured by a contact profilometer (TR200, Time group Inc., China) and recorded in µm. The measurements were done before and after acid immersion for each specimen and the mean values were used to obtain Δ*R* for each specimen.

### 2.6. Surface Topography

The surface topography was studied using a SEM (MV2300, VEGA, TESCAN, Czech Republic). One specimen of each group was scanned before and after immersion at a 250 × and 500 × magnification to determine the degree of damage on the surface after exposure to simulated gastric acid.

### 2.7. Statistical Analysis

Statistical analysis was performed with softwares (SPSS V 27 and R (4.2.2)). We used parametric tests due to normal distribution of data. Paired-sample *t* test was used to evaluate the effect of acid immersion on each parameter and independent sample *t* test was used to compare different parameters between IPS Ivocolor group and GC group. The significance level was set at *p* ≤ 0.05.

## 3. Results

### 3.1. Color Change and Translucency Parameter

Regarding color change (Δ*E*_ab_ and Δ*E*_00_), data analysis showed that acid immersion had a significant effect on color in both groups (*p*-value < 0.001) with IPS Ivocolor having a higher mean Δ*E* compared to GC group (*p*-value < 0.05).

Based on Δ*E*_ab_ the changes in color parameters, Δ*a*^*∗*^ and Δ*b*^*∗*^ were towards red and yellow coordinates in both groups, while Δ*l*^*∗*^ was towards black (darker) for GC group and towards white (lighter) in IPS Ivocolor. Detailed results are represented in Table 1.

Based on Δ*E*_00_ GC specimens become darker with an increase in saturation of the chroma, while IPS Ivocolor had become lighter with an increase in saturation of chroma, as represented in Table 2. Using pearson's correlation coefficient, Δ*E*_00_ and Δ*E*_ab_ showed a significant positive correlation with a *r*-value of 0.99.

**Figure 1:**
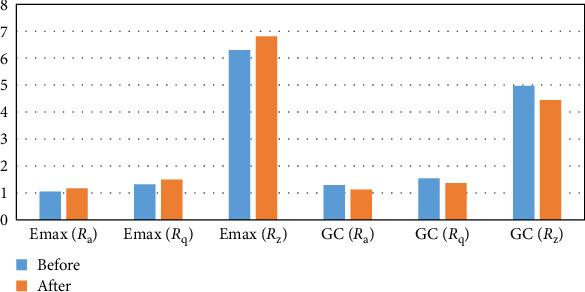
Surface roughness (*R*_a_, *R*_z_, and *R*_q_) comparison between GC and IPS Ivocolor before and after acid immersion.

Acid immersion had no statistically significant effect on TP values and there was no meaningful difference between two groups before and after immersion (Figure 1, Table 3).

### 3.2. Surface Roughness

Acid immersion resulted in a significant increase in *R*_a_ (*p*-value < 0.01), *R*_z_ (*p*-value < 0.05), and *R*_q_ (*p*-value < 0.001) values in IPS Ivocolor. IPS Ivocolor had higher *R*_z_ before immersion (*p*-value < 0.05) and after immersion (*p*-value < 0.01) than GC group (Table 4 and Figure 1). This increase in surface roughness itself has the potential to affect light reflection and color perception. Higher surface roughness and Δ*E* values in IPS Ivocolor may confirm this correlation.

### 3.3. Surface Topography

**Figure 2:**
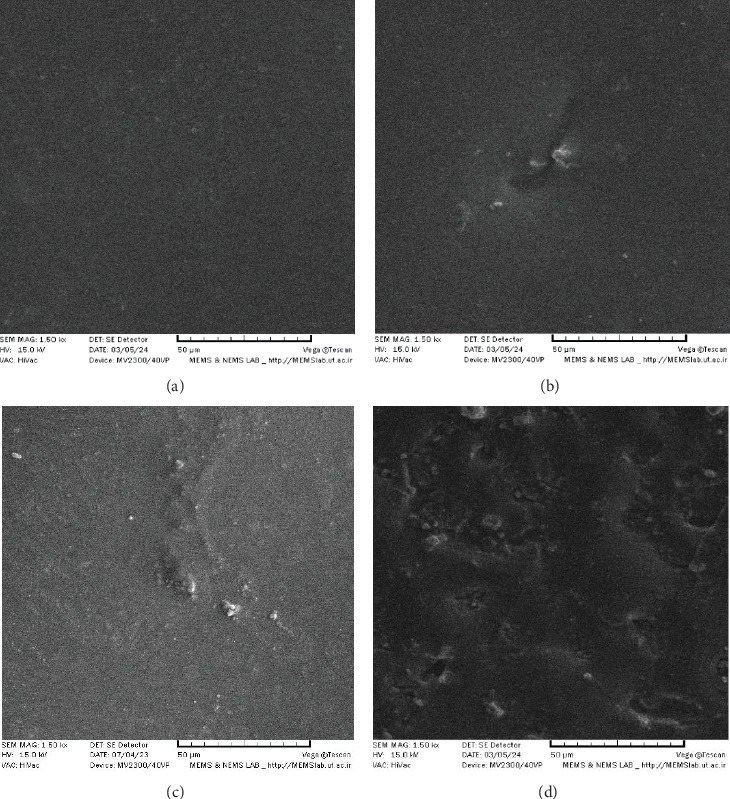
(A) GC before acid immersion, (B) GC after acid immersion, (C) IPS Ivocolor before acid immersion, and (D) IPS Ivocolor after acid immersion.

IPS Ivocolor showed a more uneven surface before aging and also a rougher surface with more fissures, pits, pores, and depressions after aging (Figure 2).

## 4. Discussion

Ceramic restorations are a popular option to rehabilitate the patients with dental erosion and among them, stained all-ceramic restorations can be an easy way to deliver a monolithic restoration without the need of a veneer layer. Since many monolithic ceramic blocks are fabricated of a single shade, external staining can be used to improve their esthetic appearance. Multiple coloring pigments in the form of oxide such as Fe, Cu, Co, Mn, and even opaque oxides such as Sn, Zn, Al, Zr, and Ti are added to low fusing glass to constitute these stains [25]. Pigments should be insoluble in water and organic solvents for color characteristics of the restorations to stay stable.

The stability of these stains in gastric acid remains unknown and to the best of our knowledge only one study by Habib et al. [19] has studied the effect of low pH on stained ceramics, in which the changes in color, translucency and surface roughness after a 16 h immersion in a 4% acetic acid was investigated based on ISO standard 6872, while many recent studies have used Hunt and MC Intyre's method [22]. Therefore, the present study was designed to evaluate the effect of simulated gastric acid on optical characteristics and surface topography of stained and glazed lithium disilicate ceramics.

Amber Mill LDS ceramic blocks and GC and IPS Ivocolor stains were used in this study. Samples were stained and glazed with brushing technique as it is known to be the most commonly used one by dental technicians. The main advantage of this method can be better characterization of the restoration as it allows multiple stain application of different shades. Sulaiman et al. [26] also stated that brushing technique has less adverse effects on mechanical and optical properties of ceramics compared to other application technique including dipping and spraying, used for different materials. About the corrosive environment, there is no clear consensus on acid concentration and Immersion time in the literature. ISO standard 6872 recommends the use of a 4% acetic acid for 16 h at 80°C, which is supposed to be equivalent to 2 years in clinic [27]. The detrimental effect of acids mainly depends on the acid concentration, immersion time and the temperature. In this study, a 0.06 M hydrochloric acid (pH = 1.2) was used based on HUNT and MC Intyre's method as used in some previous studies [4, 13, 17, 18, 22]. Also, immersion time was increased to 96 h at 37°C which is assumed to be equivalent to over 10 years of clinical exposure.

Our null hypothesis was rejected as acid changed some of the optical characteristics in both groups and surface roughness in IPS Ivocolor group.

Regarding color changes, IPS Ivocolor had a detectable but acceptable color change (0.8 < Δ*E*_00_ < 1.8 and 1.2 < Δ*E*_ab_ < 2.7). The color change for GC group was visually undetectable (Δ*E*_ab_ < 1.2 and Δ*E*_00_ < 0.8).. There was a meaningful difference between two groups. Habib et al. [19] also showed that corrosive environment significantly affects the color of stained ceramics but in an unacceptable way, considering that they have used the same stain application technique using a bristle brush but different stains (IPS Ivocolor dentin shade (SD5) and Vita Akzent [Akz 10]).

Translucency values showed no significant change which is in disagreement with Habib et al. [19] who showed that acid immersion caused a reduction in translucency of stained and glazed ceramics. There are also other studies which have evaluated the effect of acid immersion on translucency of different dental ceramics without being stained and glazed. Sulaiman et al. [13] showed that simulated gastric acid caused an increase in translucency of IPS emax (lithium disilicate) and two types of zirconia material but had no significant effect on other materials [13]. In another study by Pîrvulescu et al. [4], gastric acid didn't affect the translucency of CAD–CAM ceramics, while most of monolithic ceramics assessed in Aldamaty's study showed an increase in translucency after immersion in gastric acid [20].

According to the surface roughness assessment, IPS Ivocolor had a higher initial *R*_a_, *R*_z_, and *R*_q_ value and also a significant change after aging. Since the extrinsic stain layers can be covered by a glass layer to increase the durability of the restoration's color, as they were in this study, the increase in surface roughness can be attributed to the degradation of glaze layer. Further examination of the surface by SEM showed a more uneven surface after acid immersion especially in IPS Ivocolor group which can be due to the detrimental effect of simulated gastric acid and partial loss of the stain and glaze layer.

Totally GC stain showed more stability in corrosive environment but we should mention that these results can be attributed to numerous factors such as the application technique (brushing in the present study), the type of underlying ceramic and the compatibility between the stain and ceramic.

The main limitation of this study was that ceramic restorations are subject to complex mechanical, thermal, and chemical challenges in oral cavity, which are challenging to exactly simulate in an in vitro experiment. Another limitation is the fact that we only assessed one stain of the two brands. Since different stains have different constituents, future studies are needed to evaluate the effect of acidic environment on other stains.

## 5. Conclusion

Within the limitation of this study, low pH had a detrimental effect on the optical properties and surface texture of stained lithium disilicate blocks. Therefore, layered restorations should be considered in patients with GERD, bulimia nervosa, or any other condition exposing ceramic restorations to low pH.

## Figures and Tables

**Table 1:** The difference in Δ*a*^*∗*^, Δ*b*^*∗*^, Δ*L*^*∗*^,  and Δ*E*_ab_ between GC and IPS Ivocolor.

Outcome	IPS Ivocolor		GC		Mean difference	SE	*p*-Value	*t*
	*M*	SD	*M*	SD				
Δ*E*	2.16	1.335	0.85	0.524	1.312	0.478	<0.05	2.745
Δ*L*	0.05	1.941	−0.13	0.907	0.182	0.714	0.802	0.255
Δ*a*	0.09	0.723	0.01	0.197	0.081	0.250	0.754	0.323
Δ*b*	0.40	1.600	0.21	0.397	0.193	0.549	0.734	0.350

**Table 2:** The difference in Δ*L*_00_, Δ*C*_00_, Δ*H*_00_,  and Δ*E*_00_ between GC and IPS Ivocolor.

Outcome	IPS Ivocolor		GC		Mean difference	SE	*p*-Value	*t*
	*M*	SD	*M*	SD				
Δ*E*_00_	1.68	1.090	0.69	0.497	0.99	0.399	<0.05	2.48
Δ*L*_00_	0.04	1.697	−0.12	0.806	0.16	0.627	0.797	0.26
Δ*C*_00_	0.23	0.910	0.11	0.208	0.12	0.311	0.722	0.37
Δ*H*_00_	−0.09	0.767	−0.02	0.231	−0.07	0.267	0.808	−0.25

**Table 3:** The difference in translucency parameter before and after acid immersion between GC and IPS Ivocolor.

Variable	Mean (SD)		Mean difference	SE	*p*-Value	*t*
	IPS Ivocolor	GC				
Tp-before	13.24 (1.504)	13.60 (1.460)	−0.37	0.663	0.586	−0.55
Tp-after	13.62 (1.384)	14.12 (1.209)	−0.49	0.612	0.427	−0.81
After-before	0.30 (2.36)	0.53 (1.009)	−0.22	0.855	0.799	−0.26

**Table 4:** Surface roughness (*R*_a_, *R*_z_,  and *R*_q_) comparison between GC and IPS Ivocolor before and after acid immersion.

Time	Variable	Mean (SD)		Mean difference	SE	*p*-Value	*t*
		IPS Ivocolor	GC				
Before aging	*R*_a_	1.05 (0.207)	1.29 (0.198)	−0.24	0.094	<0.05	−2.65
	*R*_q_	1.32 (0.24)	1.54 (0.402)	−0.22	0.160	0.187	−1.38
	*R*_z_	6.30 (1.207)	4.97 (.214)	1.33	0.586	<0.05	2.27

After aging	*R*_a_	1.17 (0.162)	1.12 (0.335)	0.05	0.137	0.724	0.36
	*R*_q_	1.49 (0.211)	1.36 (0.424)	0.14	0.175	0.446	0.78
	*R*_z_	6.81 (0.924)	4.44 (1.705)	2.37	0.712	<0.01	3.34

After-before	*R*_a_	0.13 (0.075)	−0.19 (0.30)	−0.32	0.099	<0.01	−3.27
	*R*_q_	0.19 (0.087)	−0.20 (0.482)	−0.39	0.156	<0.05	−2.54
	*R*_z_	0.99 (0.897)	−0.62 (1.31)	−1.61	0.611	<0.05	−2.64

## Data Availability

The data that support the findings of this study are available from the corresponding author upon reasonable request.
